# Orphan receptor GPR50 attenuates inflammation and insulin signaling in 3T3‐L1 preadipocytes

**DOI:** 10.1002/2211-5463.13516

**Published:** 2022-12-13

**Authors:** Zhenyu Yao, Jun Meng, Jing Long, Long Li, Weicong Qiu, Cairong Li, Jian V. Zhang, Pei‐Gen Ren

**Affiliations:** ^1^ Centre for Translational Medicine Research & Development, Shenzhen Institutes of Advanced Technology Chinese Academy of Sciences Shenzhen China; ^2^ Department of Pathogenic Biology Shenzhen Center for Disease Control and Prevention China; ^3^ Department of Microbiology, School of Public Health Southern Medical University Guangzhou China; ^4^ Center for Energy Metabolism and Reproduction, Shenzhen Institutes of Advanced Technology Chinese Academy of Sciences Shenzhen China

**Keywords:** diabetes mellitus type 2, GPR50, insulin signaling, IRS1, PPAR‐γ

## Abstract

Type 2 diabetes (T2DM) is characterized by insulin secretion deficiencies and systemic insulin resistance (IR) in adipose tissue, skeletal muscle, and the liver. Although the mechanism of T2DM is not yet fully known, inflammation and insulin resistance play a central role in the pathogenesis of T2DM. G protein‐coupled receptors (GPCRs) are involved in endocrine and metabolic processes as well as many other physiological processes. GPR50 (G protein‐coupled receptor 50) is an orphan GPCR that shares the highest sequence homology with melatonin receptors. The aim of this study was to investigate the effect of GPR50 on inflammation and insulin resistance in 3T3‐L1 preadipocytes. GPR50 expression was observed to be significantly increased in the adipose tissue of obese T2DM mice, while GPR50 deficiency increased inflammation in 3T3‐L1 cells and induced the phosphorylation of AKT and insulin receptor substrate (IRS) 1. Furthermore, GPR50 knockout in the 3T3‐L1 cell line suppressed PPAR‐γ expression. These data suggest that GPR50 can attenuate inflammatory levels and regulate insulin signaling in adipocytes. Furthermore, the effects are mediated through the regulation of the IRS1/AKT signaling pathway and PPAR‐γ expression.

## Graphical abstract

Inflammation and insulin resistance play a central role in the pathogenesis of T2DM. G protein‐coupled receptors are involved in endocrine and metabolic processes. Our study found that a receptor called GPR50 can attenuate inflammatory levels and regulate insulin signaling in adipocytes. Furthermore, the effects are mediated through the regulation of the IRS1/AKT signaling pathway and PPAR‐γ expression.

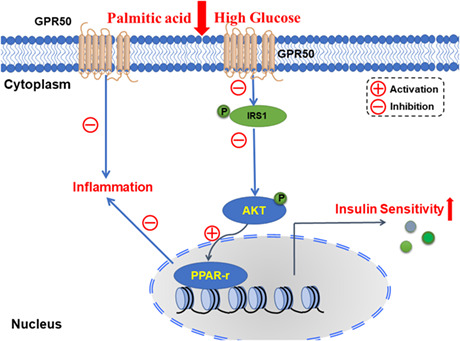

**Abbreviations**
FFA: free fatty acids
HFD: high‐fat diet
HSL: hormone‐sensitive lipase
IR: insulin resistance
IRS: insulin receptor substrate
MCP‐1: monocyte chemoattractant protein 1
PA: palmitic acid
PI3K: phosphatidylinositol 3‐kinase
PPAR‐γ: peroxisome proliferator‐activated receptor‐γ
T2DM: type 2 diabetes mellitus

Type 2 diabetes (T2DM) is a metabolic disease that is widely prevalent worldwide. It is characterized by insulin secretion deficiencies and systemic insulin resistance (IR) in adipose tissue, skeletal muscle, and the liver [1]. Although the mechanism of T2DM is not yet fully known, inflammation and insulin resistance play a central role in the pathogenesis of T2DM.

According to a recent analysis, the role of adipose tissue is crucial in the progression of diabetes. T2DM increases the mass of visceral adipose tissue [2] and results in the secretion of various inflammatory cytokines such as interleukin (IL)‐1β, monocyte chemoattractant protein 1 (MCP‐1), and IL‐6, which trigger chronic, low‐grade inflammation [3]. In a proinflammatory environment, insulin signaling pathways are activated, leading to decreased insulin sensitivity in cells. Ample evidence suggests that inflammatory mediators directly inhibit insulin signaling, with c‐Jun N‐terminal kinase (JNK), an inhibitor of nuclear factor kappa‐B kinase beta (IKKβ) and protein kinase C γ (PKCγ) blocking insulin signaling by phosphorylating insulin receptors and insulin receptor substrate (IRS) proteins at inhibitory sites [4, 5]. Nuclear factor kappa‐B (NF‐κB) can inhibit the expression of insulin signaling pathway components, including IRS1 and protein kinase B (AKT/PKB) [6, 7].

Multiple metabolic pathways are responsible for insulin resistance in obesity and T2DM [8]. Insulin signaling is thought to be regulated by the insulin receptor and its substrates phosphatidylinositol 3‐kinase (PI3K) and AKT/protein kinase B (PKB) [9, 10]. Insulin signaling is composed of two signaling pathways related to the insulin receptor. The phosphatidylinositol 3‐kinase (PI3K)‐AKT pathway and the Ras‐mitogen‐activated protein kinase (MAPK) pathway, which also interacts with the PI3K‐AKT pathway [9, 10]. Both can be activated by the insulin/insulin receptor substrate‐1 (IRS1) system in insulin resistance. According to the available findings, phosphorylation of IRS1 is strongly correlated with insulin resistance [11].

Adipose tissue is an important initial site of IR, and peroxisome proliferator‐activated receptor‐γ (PPAR‐γ) has a major influence on the differentiation of adipose tissue, macrophage polarization, and the secretion of adipogenic factors [12]. Moreover, PPAR‐γ is an important regulator of lipid metabolism and insulin sensitivity [12]. Activated PPAR‐γ heterodimerizes with retinoid X receptor‐α (RXR‐α) to maintain glucose homeostasis through direct regulation of IRS1 and glucose transporter type 4 (GLUT4) [13]. The loss of PPAR‐γ in skeletal muscle and severe IR has been demonstrated [14].

G protein‐coupled receptors (GPCRs) are involved in endocrine and metabolic processes as well as many other physiological processes. Many medicinal drugs, including glycolipid metabolism drugs, target GPCRs [15]. GPR50, also known as H9 or melatonin‐related receptor, the full‐length gene, encodes a protein of 617 amino acids, it is located on X chromosome Xq28 [16]. Because GPR50 does not bind to melatonin or any other known endogenous or synthetic ligand, it is listed as an orphan receptor. GPR50 is an orphan GPCR that shares the highest sequence homology with melatonin receptors [17]. GPR50 is highly expressed in the hypothalamus, pituitary gland, and locus coeruleus, which play critical roles in the regulation of stress and anxiety‐related disorders. GPR50 has been introduced as a new target for the treatment of major CNS diseases, major depression, and bipolar disorder [18, 19]. GPR50 has been shown to be an important regulator of energy metabolism in GPR50 knockout mice [20]. A sequence variant study suggested that GPR50 is related to mental disorders [21] and altered lipid metabolism [22].

## Materials and methods

### Materials

An antibody against IRS1 (sc‐8038, 1 : 1000) was purchased from Santa Cruz Biotechnology (Santa Cruz, CA, USA). Antibodies against phospho‐IRS1 (Ser612; 3193S, 1 : 1000), phospho‐AKT (Ser473; 4060S, 1 : 1000), AKT (4685S, 1 : 1000), β‐actin (4970S, 1 : 1000), GAPDH (5174S, 1 : 1000), and PPAR‐γ (2435S, 1 : 1000) were purchased from Cell Signaling Technology (Danvers, MA, USA). GPR50 (19762‐1‐AP, 1 : 1000) antibody was purchased from Proteintech (Rosemont, IL, USA).

### Cell culture

The American Type Tissue Culture Collection provided the mouse preadipocyte 3T3‐L1 cell line (Manassas, VA, USA). Cells were maintained in high‐glucose Dulbecco's modified Eagle's medium (DMEM; Invitrogen, Carlsbad, CA, USA) and 10% fetal bovine serum (FBS, GIBCO, Carlsbad, CA, USA) at 37 °C in a 5% CO_2_ incubator. In treatment experiments, 3T3‐L1 cells were incubated with 16.7 mm glucose or 0.25 mm palmitate for 48 h to mimic obese adipocytes [23, 24].

### Differentiation of 3T3‐L1 adipocytes

Mouse 3T3‐L1 preadipocytes were grown in DMEM with 10% FBS. Two days after reaching confluence, the cell medium was modified into a differentiation medium (StemPro™ Adipogenesis Differentiation Kit, Invitrogen). The induction medium was replaced every 3 days until the 3T3‐L1 cells had differentiated into mature adipocytes.

### Mouse model

Eight‐week‐old C57BL/6J male mice were obtained from Guangdong Medical Laboratory Animal Center (Guangdong, China). The mice were randomly divided into two groups after 1 week of adaptation: the HFD group (*n* = 3) and the chow group (*n* = 3). Mice were fed a high‐fat diet (45% fat, Research diet D12451, New Jersey) or chow diet (4% fat, KEAO XIELI, Tianjin, China) *ad libitum* for 15 weeks before the symptoms of the mice were characteristic of T2DM (Fig. S1). Body weight was assessed every 2 weeks. Animal care was in compliance with the Guide for the Care and Use of Laboratory Animals of Guangdong Province. All the procedures were under the supervision of and approved by the Ethics Committee for Animal Research, Shenzhen Institutes of Advanced Technology, Chinese Academy of Sciences (Approval Number: SIAT‐IRB‐170401‐YGS‐RPG‐A0312‐01).

### RNA extraction

Epididymal fat tissues were isolated from the mice. RNA samples were collected from the adipose tissue using TRIzol reagent (Invitrogen) and were validated with the Agilent Array platform for microarray assay and real‐time PCR analysis.

### Microarray analysis

The microarray study was performed using GMINIX (Shanghai, China) with Mouse Transcriptome Array 1.0 (Affymetrix, Santa Clara, CA, USA). This microarray targets approximately 114 000 protein‐coding transcripts. We purified RNA from epididymal adipose tissues from three HFD and three chow mice and used a random priming process to transcribe the RNA into complementary DNA (cDNA). In a GeneChip® Hybridization Oven 645, cDNA was fragmented and biotinylated, and 5.5 μg of cDNA was hybridized to the GeneChip Mouse Transcriptome Array 1.0. The arrays were screened with the Gene Array Scanner 3000 7G (Affymetrix) after hybridization and washing. All data were analyzed with the Robust Multichip Analysis (RMA) algorithm using Affymetrix default analysis settings and global scaling as a normalization process.

### Quantitative real‐time PCR

Total RNA was extracted with TRIzol reagent from cells and mouse adipose tissue (Invitrogen). Reverse transcription and RT–PCR were performed using an RT–PCR Kit (Takara, Otsu, Japan). For Q‐PCR, a Light Cycler (Roche, Basel, Switzerland) and SYBR quantitative real‐time PCR (Takara) kit were used. The endogenous control was GAPDH. Table 1 presents the PCR primers used for mRNA quantitation; these were synthesized by Sangon Biotech (Shanghai, China).

**Table 1:** The primers of target genes and GAPDH.

Target gene name	Primer
GPR50	F: AGCGGTTTGGACTCACTGAA
	R: CCAAGACAGCCTTGACCAGT
IL‐6	F: TAGTCCTTCCTACCCCAATTTCC
	R: TTGGTCCTTAGCCACTCCTTC
MCP‐1	F: TTAAAAACCTGGATCGGAACCAA
	R: GCATTAGCTTCAGATTTACGGGT
IL‐1beta	F: GCAACTGTTCCTGAACTCAACT
	R: ATCTTTTGGGGTCCGTCAACT
PPAR‐γ	F: CTGGCCTCCCTGATGAATAA
	R: CGCAGGTTTTTGAGGAACTC
GAPDH	F: TGTGTCCGTCGTGGATCTGA
	R: CCTGCTTCACCACCTTCTTGAT

### Enzyme‐linked immunosorbent assay

GPR50 knockout 3T3‐L1 or control cells were incubated with 16.7 mm glucose or 0.25 mm palmitate for 48 h as described above. The supernatants were collected and centrifuged at 1509 *g* for 5 min. The concentrations of IL‐6 (Dakewe, Shenzhen, China), MCP‐1 (Sino Biological, Beijing, China), and IL‐1β (R&D Systems, Minneapolis, MN, USA) were assayed with ELISA kits according to the manufacturers' instructions. Quantitative data are presented as average concentrations in pg·mL^−1^.

### Construction of the GPR50 knockout cell line

To establish a stable GPR50 knockout 3T3‐L1 cell line, GPR50 small guide RNAs (sgRNAs) were designed using an online CRISPR design tool (http://crispr.mit.edu). The sgRNA sequence was as follows: GTTTCAGAGCTATGCTGGAAACAGCATAGCAAGTTGAAATAAGGCTAGTCCGTTATCAACTTGAAAAAGTGGCACCGAGTCGGTGCT. For further analysis, the sequences were synthesized and annealed to form a gRNA duplex. BsmB I digested the lentiviral vector (OBiO, Shanghai, China), resulting in sticky ends. The phosphorylated and annealed sgRNA was ligated to the lentiviral vector. Sequencing confirmed the correct clone, and the high‐purity plasmid was extracted. Shanghai OBiO Technology Co., Ltd. completed the lentiviral packaging. The 3T3‐L1 cell suspension was added to each well of a six‐well plate at a density of 5 × 10^4^ cells·mL^−1^, and lentivirus was added to each well at the proper density. For monoclonal cell screening, 2 μg·mL^−1^ puromycin was introduced after 72 h of infection. Forty‐eight hours later, the screened and selected cells were divided and seeded into 96‐well plates at 1 cell per well using flow cytometry. After approximately 2 weeks, single‐cell colonies were obtained; a single‐cell pellet was picked up with a microscope and inoculated into a new 24‐well plate to begin the culture. Empty lentivector was used as a negative control. Cells infected with knockout lentivector (KO) were designated KO cells, and the cells with empty lentivector infection were designated control cells. After selection, the efficiency of infection was verified by a western blot.

### Insulin stimulation

GPR50 knockout 3T3‐L1 or control cells were seeded in a 6‐well plate. Two days after reaching confluence, cells were serum‐starved in DMEM for 4 h and then washed three times with PBS. Incubation of the cells in DMEM with 100 nm insulin or DMEM alone for 30 min. Finally, cells were washed three times and protein was extracted for western blot detection.

### Western blot analysis

The 3T3‐L1 cells were lysed with a cell lysis buffer (Cell Signaling Technology) for western blot analysis, and the extracted protein concentration was determined with a BCA protein assay kit (Pierce, Rockford, IL, USA). Protein (30 μg) was subjected to 4–10% SDS/PAGE and transferred to a PVDF membrane (Amersham Biosciences, Buckinghamshire, UK). The membranes were then blocked for 2–3 h at room temperature with 5% (w/v) BSA. The membranes were incubated overnight at 4 °C with the indicated antibodies and then for 1 h with horseradish peroxidase‐conjugated secondary antibodies, followed by visualization with a chemiluminescence system (ECL, Amersham Biosciences) on a ChemiDoc MP System (Bio‐Rad, BioRad, Richmond, CA, USA). Washings were performed between incubations.

### Statistical analysis

Quantitative real‐time PCR results are presented as the relative quantity to the control group, using β‐actin as an internal reference gene. Band densities were corrected for background and then normalized to the control (IRS1 for p‐IRS1/AKT for p‐AKT/β‐actin or GAPDH for PPAR‐γ) signal of the same lane in the western blot experiment. ELISA experimental results are normalized as the relative quantity to the control group. All data are presented as the mean ± SEM, and the differences between the two groups were measured using a *t* test. More than two groups were compared using a two‐way ANOVA with *P* < 0.05 indicating statistical significance. All statistical analyses were conducted using graphpad prism 7 (GraphPad Software, San Diego, CA, USA). At least three biological replicates of each experiment were performed and analyzed.

## Results

### GPR50 as a novel candidate target in obesity‐T2DM animal adipose tissue

**Fig. 1:**
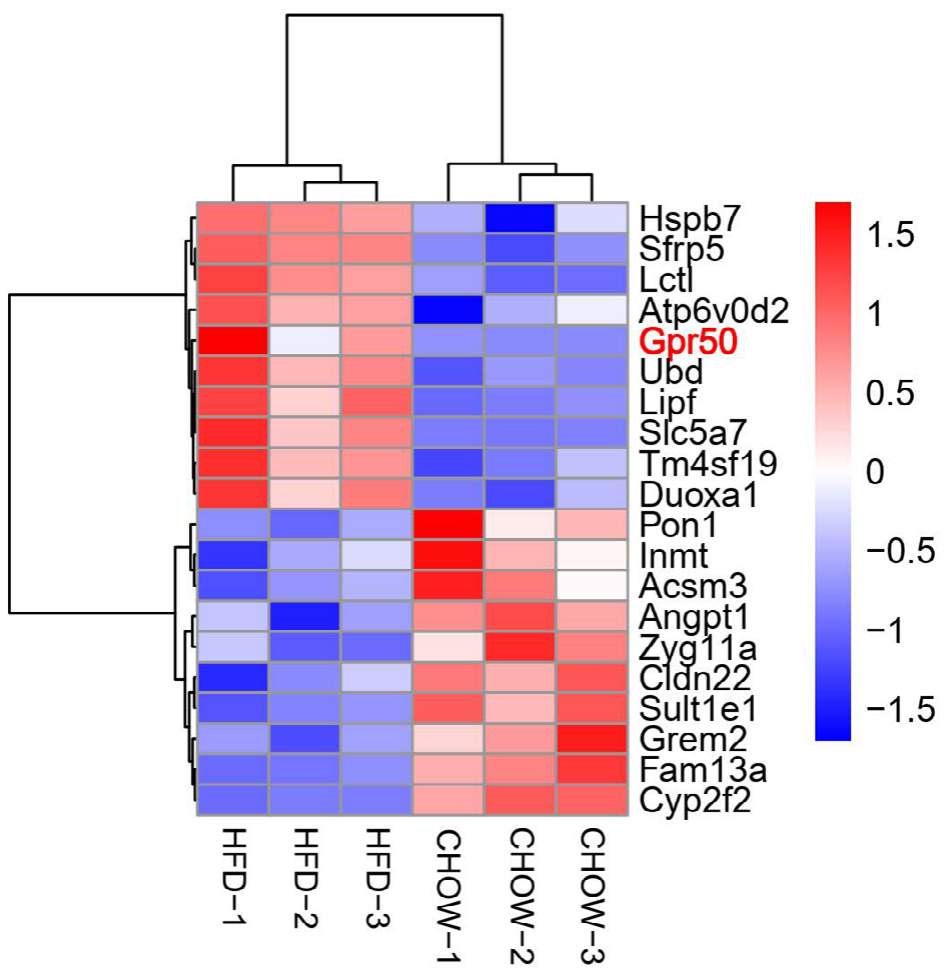
Hierarchical clustering analysis of most 20 differentially expressed genes in chow mice and HFD mice. Differentially expressed mRNAs in CHOW (*n* = 3) and HFD mice (*n* = 3). Red and blue color indicates up‐regulated and down‐regulated transcripts, respectively.

To systemically screen new targets from adipose tissue in obese T2DM mice, we compared two groups of male C57BL/6J mice fed a standard chow diet or HFD (*n* = 3) using transcriptome analyses. The microarray information is available in the Gene Expression Omnibus (GSE100028). The results of our previously published paper showed that HFD mice exhibited obesity and T2DM symptoms [25]. As shown in Fig. 1, we selected the 20 most significantly differentially expressed genes between the chow and HFD groups. Among these genes, we found that the expression of an orphan GPCR designated GPR50 (3.73 ± 1.291 *P* < 0.05) was significantly increased in the adipose tissue of the HFD group compared with the chow group. Given the importance of GPCRs for discovering new targets for T2DM treatment and our experiment with GPCR, we investigated the effect of GPR50 on T2DM.

### Expression profiles of GPR50 in adipose tissue and differentiated 3T3‐L1 cell lines

**Fig. 2:**
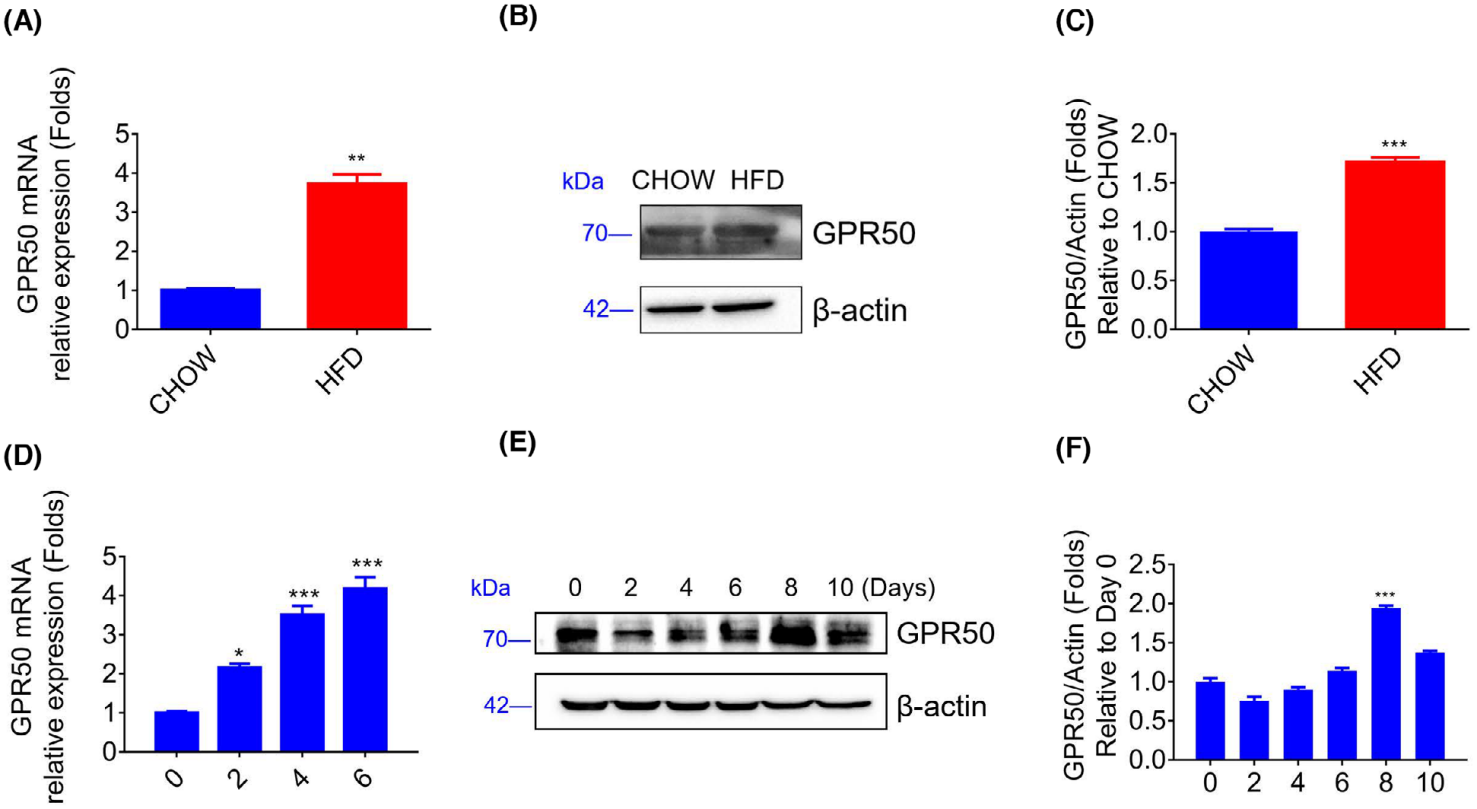
Analysis of GPR50 expression in mouse adipose tissue and the 3T3‐L1 cell line. Epididymal fat pads of CHOW and HFD C57BL/6J mice were lysed, and GPR50 mRNA expression was detected by Q‐PCR (A). Protein expression of GPR50 was evaluated by a western blot (B, C). 3T3‐L1 preadipocytes differentiated into adipocytes for more than 6 days. GPR50 mRNA expression was detected by Q‐PCR (D), and GPR50 protein levels were evaluated by western blotting (E, F). Western blots are shown along with relative densities determined using imagej software. Data were analyzed using *t* test. The results are representative of three independent experiments. Values in bar graphs are the mean ± SEM. *P < 0.05; **P < 0.01; ***P < 0.001 compared with day 0.

In epididymal adipose tissue, we confirmed that GPR50 expression increased in HFD‐fed mice at both the mRNA and protein levels compared with those in chow‐fed mice, which was consistent with the microarray results (Fig. 2A–C). We then studied GPR50 expression in 3T3‐L1 preadipocytes during lipogenic differentiation. The results revealed that after 2 days of adipogenic differentiation, the expression of GPR50 increased in a time‐dependent manner and peaked at 8 days (Fig. 2D–F). These findings show that GPR50 may be involved in adipogenic differentiation.

### Construction of the GPR50 knockout 3T3‐L1 cell line

**Fig. 3:**
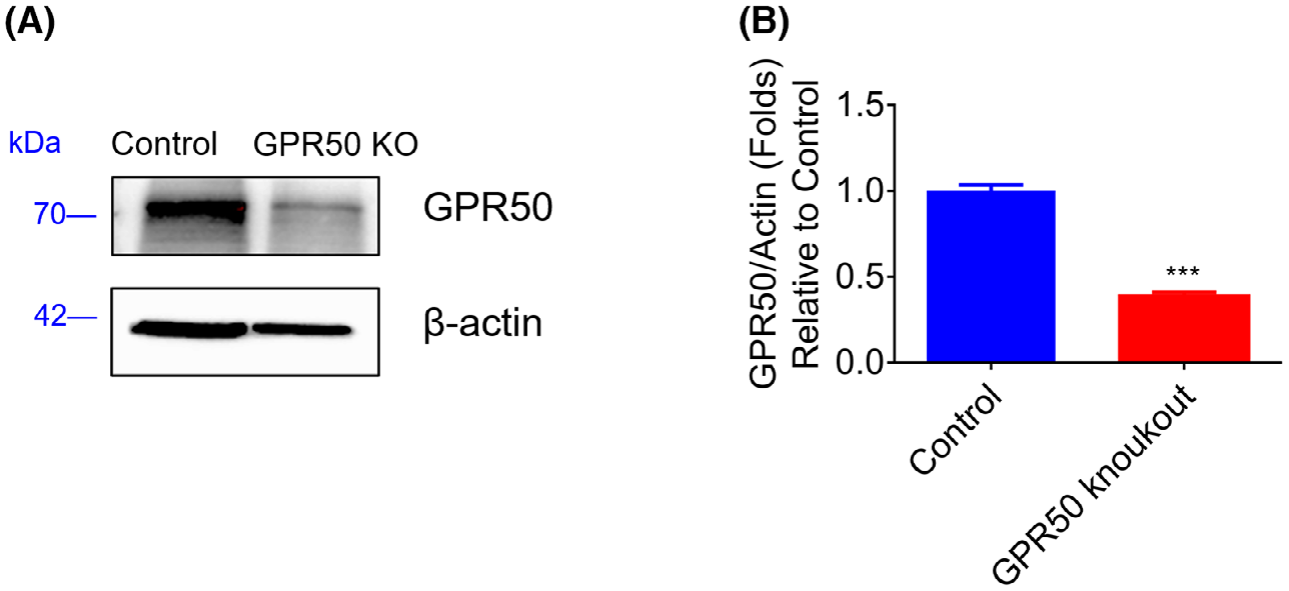
Analysis of GPR50 expression in the GPR50 knockout 3T3‐L1 cell line. The protein expression level of GPR50 was evaluated by western blotting in the GPR50 knockout 3T3‐L1 cell line (A, B). Western blots are shown along with relative densities determined using imagej software. Data were analyzed using *t* test. The results are representative of three independent experiments. Values in bar graphs are the mean ± SEM. ***P < 0.001 compared with control.

To further investigate the function of GPR50 in T2DM, we constructed a GPR50 knockout cell line using the CRISPR/Cas9 gene editing system. The full‐length sequence of GPR50 was obtained from the GenBank database. According to the general principles of gRNA design, single‐guide RNAs (sgRNAs) targeting GPR50 genes were built using online software for CRISPR design (http://crispr.mit.edu). Methods and procedures have shown the sgRNA sequence. The transfer vector was successfully constructed by combining sgRNA after annealing connection. To improve transfection efficiency, the transfer vector was packaged in lentiviruses and successfully transfected into 3T3‐L1 cells. The stable cell lines with the GPR50 gene knockout were screened using puromycin and confirmed by western blotting, as shown in Fig. 3.

### Inflammation increased in the GPR50 knockout 3T3‐L1 cell line

**Fig. 4:**
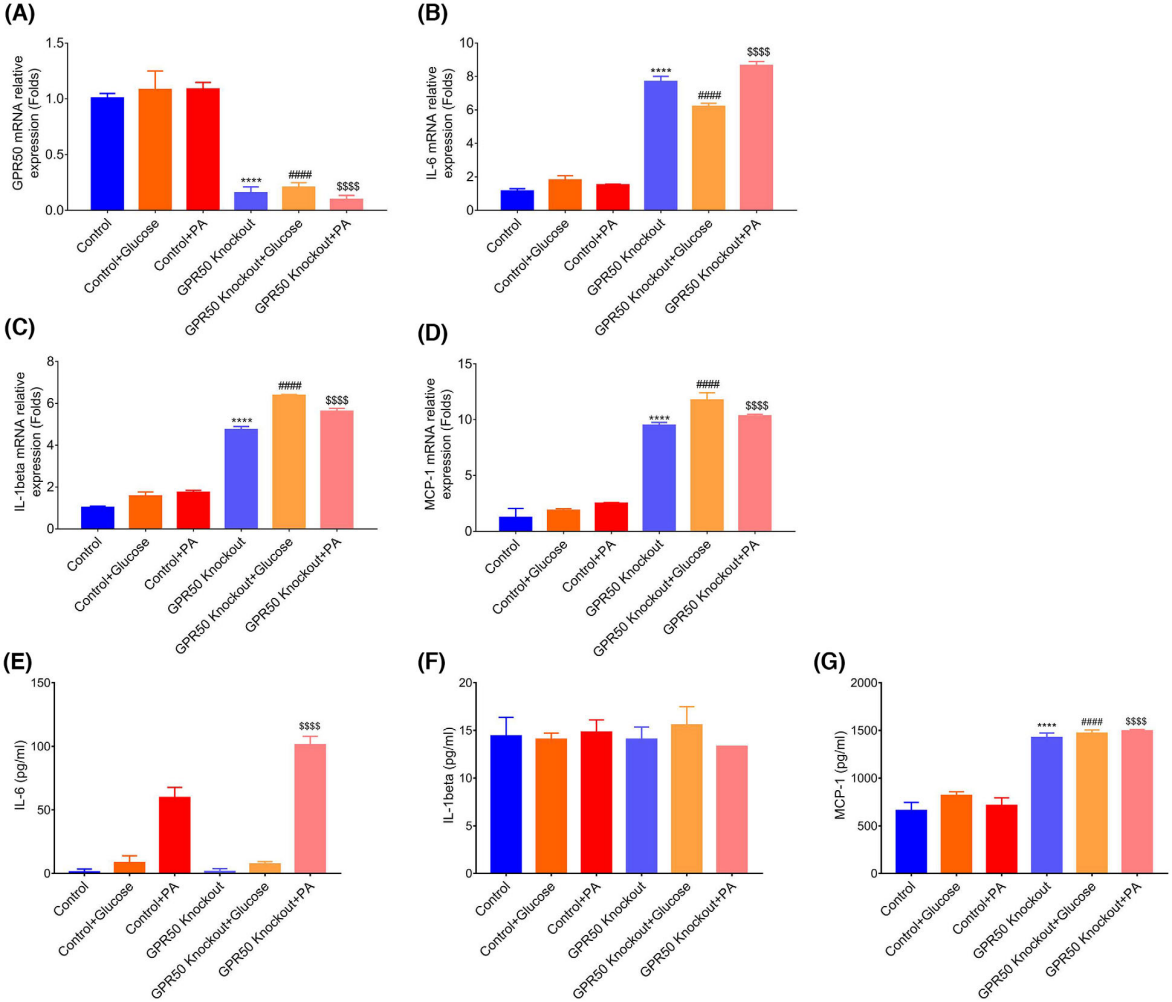
GPR50 inhibits inflammation in the 3T3‐L1 cell line. Two cell lines were stimulated with high glucose (16.7 mm) and PA (0.25 mm) for 48 h. The relative mRNA expression levels of GPR50 (A), IL‐6 (B), IL‐1β (C), and MCP‐1 (D) were analyzed by Q‐PCR; IL‐6 (E), IL‐1β (F), and MCP‐1 (G) were analyzed by ELISA. Data were analyzed using a two‐way ANOVA followed by Dunnett's multiple comparisons test. The results are representative of three independent experiments. Values in bar graphs are the mean ± SEM. ****P < 0.0001 compared with control, ####*P* < 0.0001 compared with control + glucose, $$$$*P* < 0.0001 compared with control + PA.

Obesity is related to the pathogenesis of IR in adipose tissue due to chronic low‐grade inflammation. Selected markers of adipose inflammation in the GPR50 knockout 3T3‐L1 cell line were measured. We used high glucose and palmitic acid (PA) to simulate high‐fat diet stimulation. As shown in Fig. 4A–D, at the mRNA level, the expressions of the proinflammatory cytokines IL‐6, MCP‐1, and IL‐1β, which are associated with IR, were significantly higher in GPR50 knockout cells than those in control cells transfected with the control plasmid. Moreover, we analyzed the protein secretion of IL‐6, MCP‐1, and IL‐1β in the cell supernatant using ELISA (Fig. 4 E–G). The results were not fully consistent with the Q‐PCR results. IL‐6 protein secretion increased with palmitic acid (PA) stimulation; MCP‐1 was significantly higher in GPR50 knockout cells stimulated with high glucose and palmitic acid (PA). The GPR50 overexpression experiment (Fig. S2) also demonstrated that GPR50 could inhibit MCP‐1 expression. This suggests that MCP‐1 may be more important than other protein secretions. All these findings indicated that GPR50 could improve adipose tissue inflammation induced by high glucose and PA.

### Activation of insulin signaling in GPR50 knockout 3T3‐L1 cells

**Fig. 5:**
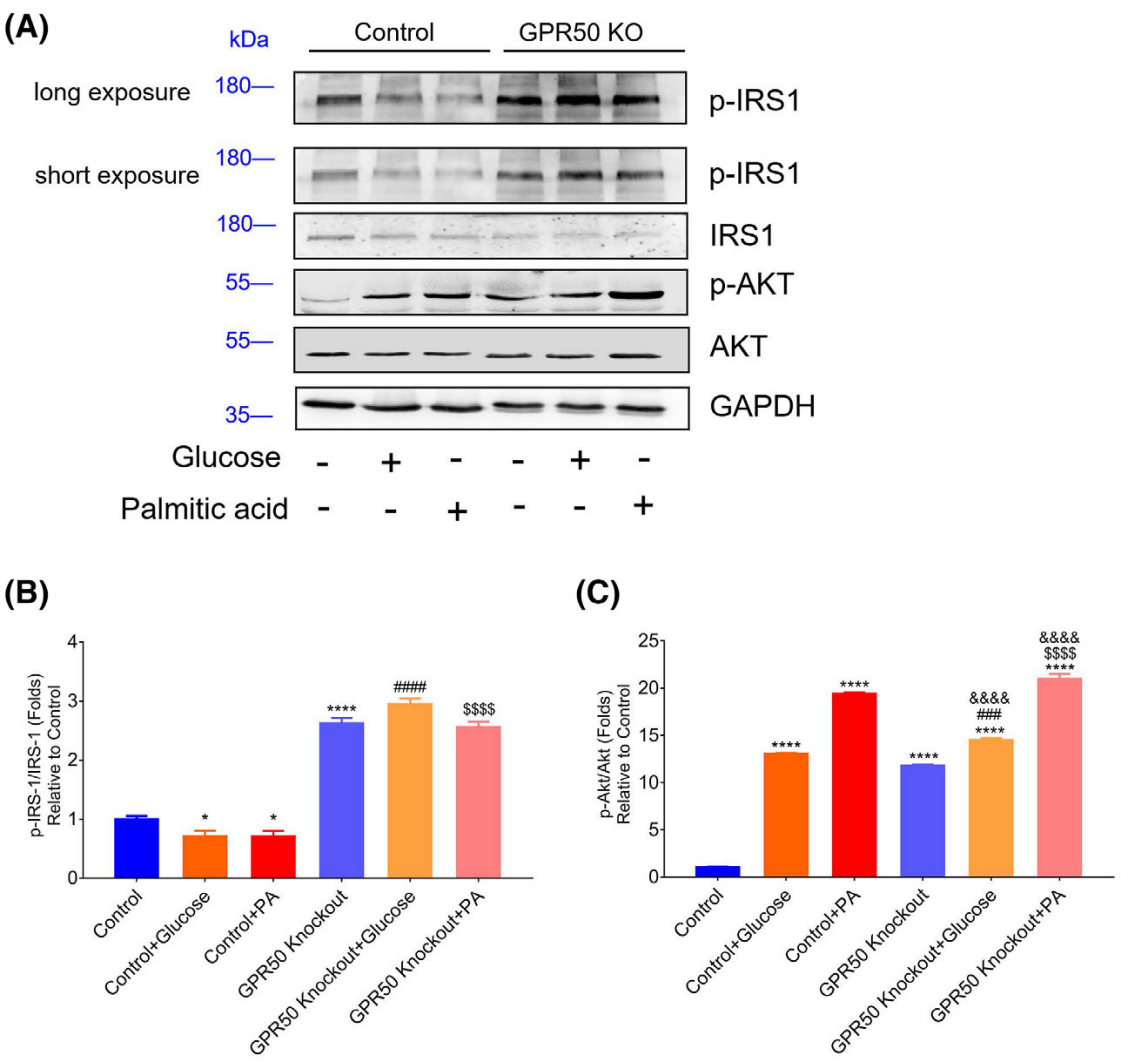
Deficiency of GPR50 activated the insulin signaling pathway in the 3T3‐L1 cell line. The protein expression of p‐IRS1, IRS1, p‐AKT, AKT, and GAPDH was analyzed by western blotting (A). The relative expressions of p‐IRS1/IRS1 (long exposure) (B) and p‐AKT/AKT (C) are representative. Western blots are shown along with relative densities determined using imagej software. Data were analyzed using a two‐way ANOVA followed by Dunnett's multiple comparisons test. The results are representative of three independent experiments. Values in bar graphs are the mean ± SEM. *P < 0.05, ****P < 0.0001 compared with control; ###*P* < 0.001, ####*P* < 0.0001 compared with control + glucose; $$$$*P* < 0.0001 compared with control + PA; and &&&&*P* < 0.0001 compared with GPR50 knockout.

IR is a pathological condition frequently linked to T2DM. GPCRs participate in the development of IR, which can lead to T2DM induced by obesity [26]. To evaluate the effect of GPR50 on the insulin signaling pathway, the protein levels of p‐IRS1/IRS1 and p‐AKT/AKT in GPR50 knockout 3T3‐L1 cells pretreated with glucose or PA were analyzed by western blotting. IRS1 phosphorylation was significantly enhanced in the GPR50‐deficient 3T3‐L1 cell line (Fig. 5A,B). Although high glucose and PA induced the phosphorylation of AKT, the GPR50‐deficient 3T3‐L1 cell line still had significantly higher p‐AKT levels than the control cells (Fig. 5A–C). High glucose and PA treatments led to an interruption of the insulin pathway by reducing p‐IRS1/IRS1, and this effect was reversed when GPR50 was removed (Fig. 5A,B). However, unlike p‐IRS1/IRS1, glucose and PA significantly increased p‐AKT/AKT levels in 3T3‐L1 cells and were high even in GPR50 knockout 3T3‐L1 cells under these treatments. These results suggest that GPR50 may improve insulin signaling with high glucose and PA activation by reducing activation of the IRS1 pathway. However, it is possible to significantly increase AKT phosphorylation through other gPR50‐related or unrelated pathways that have not been investigated (Fig. 5A,C). To further evaluate the effect of GPR50 on insulin signaling, we performed the insulin signaling test with insulin stimulation. As shown in Fig. 7A–D, insulin stimulation increased the phosphorylation levels of AKT and insulin receptor substrate (IRS)1; however, the protein expressions of p‐IRS‐1 and p‐AKT were significantly decreased in the 3T3‐L1 cell line with GPR50 knockout (Fig. 7A–D). In contrast to PA and high glucose stimulation, p‐IRS‐1/IRS‐1, and p‐AKT/AKT levels were significantly increased in 3T3‐L1 cells and significantly decreased in 3T3‐L1 cells with GPR50 knockout. These results also suggest that GPR50 may be able to inhibit insulin signaling activated by insulin through the IRS‐1/AKT pathway, although PA and insulin stimulate insulin signaling in opposite ways.

### GPR50 enhanced the expression of the transcription factor PPAR‐γ

**Fig. 6:**
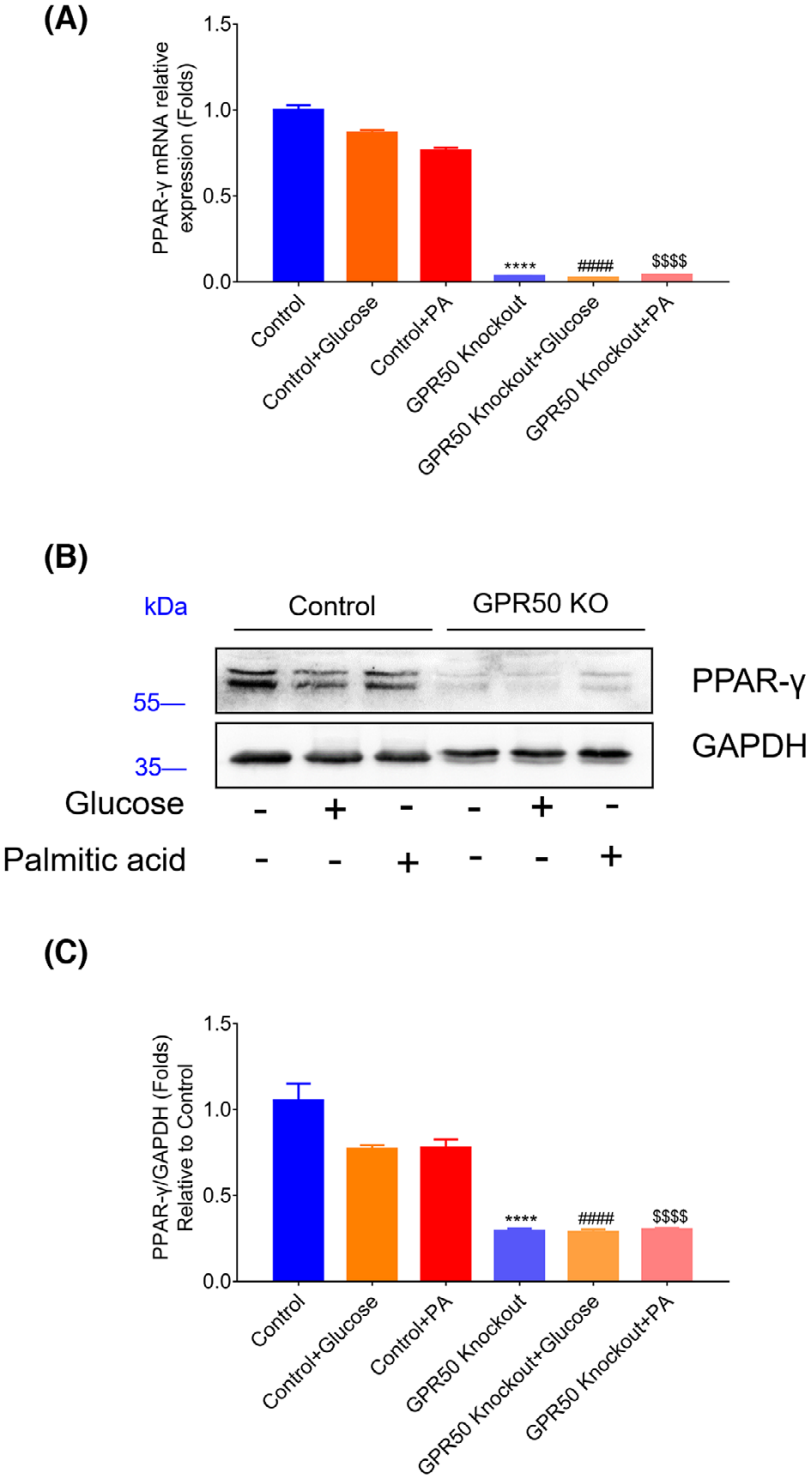
GPR50 deficiency suppressed PPAR‐γ expression in the 3T3‐L1 cell line. The mRNA expression level of PPAR‐γ was analyzed by Q‐PCR (A). The protein level of PPAR‐γ was analyzed by a western blotting (B, C). Western blots are shown along with relative densities determined using imagej software. Data were analyzed using a two‐way ANOVA followed by Dunnett's multiple comparisons test. The results are representative of three independent experiments. Values in bar graphs are the mean ± SEM. ****P < 0.0001 compared with control, ####*P* < 0.0001 compared with control + glucose, $$$$*P* < 0.0001 compared with control + PA.

**Fig. 7:**
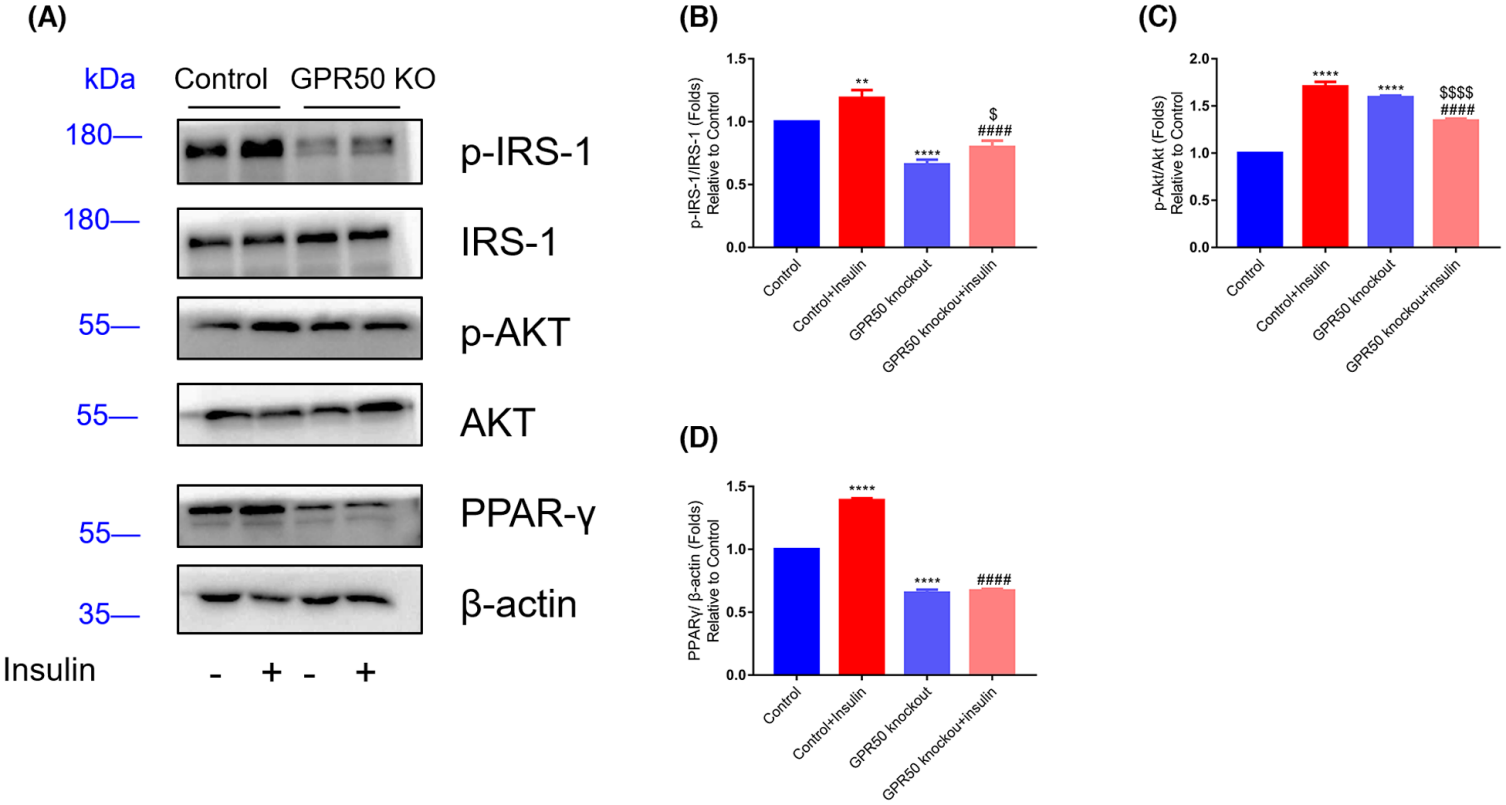
Deficiency of GPR50 activated insulin signaling pathway in 3T3‐L1 cell line with 100 nm insulin stimulates. The proteins expression of p‐IRS‐1(Ser612), IRS‐1, p‐Akt (Ser473), Akt, and β‐Actin were analyzed by western blot (A–D). Relative expression of p‐IRS‐1/IRS‐1, p‐Akt/Akt, and PPAR‐γ/β‐Actin representative. Western blots are shown along with relative densities, determined using imagej software. Data were analyzed using a two‐way ANOVA followed by Dunnett's multiple comparisons test. Values in bar graphs are means ± SEM. **, ****: *P* < 0.01 and *P* < 0.0001 compared to control; ####: *P* < 0.0001 compared to control + insulin; $, $$$$: *P* < 0.05, *P* < 0.0001 compared to GPR50 knockout.

PPAR‐γ is an essential transcription factor expressed mainly in mammalian adipose tissue and other tissues for cell differentiation. PPAR‐γ is involved in modulating insulin sensitivity. As shown in Fig. 6, GPR50 deficiency inhibited PPAR‐γ expression at the mRNA and protein levels and was related to activating insulin signaling. However, treatment with high levels of glucose or PA had no effect on the mRNA or protein expression levels of PPAR‐γ in control 3T3‐L1 cells. More interestingly, with insulin stimulation, we also found that PPAR‐γ expression was inhibited in GPR50 knockout cell lines (Fig. 7A–D). These results showed that GPR50 could still regulate the expression of PPAR‐γ under insulin stimulation, and the regulation was consistent with PA and high glucose stimulation, which were conducive to improving insulin signaling.

## Discussion

GPR50 was screened in HFD‐induced mouse adipose tissue using gene microarray technology, suggesting that it may be functional in the metabolism and development of T2DM. Previous studies have shown that GPR50 is closely related to energy expenditure and feeding behavior [20]. GPR50 knockout mice have higher metabolic rates and less fat accumulation and are partially resistant to diet‐induced obesity. These data suggest that GPR50 is a regulator of energy metabolism [20].

T2DM and IR are related to low‐grade inflammation in adipose tissue [3]. As shown in Fig. 4, we used high glucose and palmitic acid (PA) to simulate high‐fat diet stimulation. We found that GPR50 could inhibit IL‐6, IL‐1β, and MCP‐1 at the mRNA level but not at the protein level. IL‐6 protein secretion increased with palmitic acid (PA) stimulation; the protein concentration of IL‐6 was 50–100 pg·mL^−1^, whereas MCP‐1 was significantly enhanced by high glucose and palmitic acid (PA) stimulation, and the protein concentration reached 1500 pg·mL^−1^. Hypertrophied adipocytes have been found to secrete large quantities of MCP‐1, which functions as a chemoattractant that enhances macrophage infiltration into adipose tissue in obese mice and humans [27]. In fact, MCP‐1 is secreted by adipocytes in crown‐like structures and stimulates the proliferation of surrounding adipose tissue macrophages (ATMs) [28]. MCP‐1 secreted by adipose tissue contributes to macrophage infiltration in diet‐induced obese and insulin‐resistant animals [29]. After acute high saturated fatty acid stimulation, preadipocytes have a heightened inflammatory cytokine response, particularly MCP‐1 expression. Furthermore, preadipocytes recruit macrophages via MCP‐1 in adipose tissue and produce inflammatory responses [30, 31]. The increased production of MCP‐1 by adipocytes might contribute to a proinflammatory state. However, our results suggest that GPR50 significantly inhibits the secretion of MCP‐1 and alleviates the proinflammatory state of adipocytes. In recent years, there has been increasing evidence that inflammation is an inherent cause of IR during the development of obesity and T2DM. Studies in obesity and T2DM have revealed the correlation of proinflammatory signaling pathways and insulin sensitivity [32, 33, 34]. Some GPCRs have been shown to participate critically in certain processes of inflammation such as GPR84 and GPR91, both of which play an important regulatory role in inflammation in immune cells [35, 36, 37, 38]. However, there are still many unknown mediators that are involved in regulating inflammation in adipocytes. Finding new mediators associated with low‐grade adipocyte inflammation that are sufficient to induce IR in obese and T2DM individuals is important.

From this study, it appears that GPR50 has an inhibitory effect on inflammation in adipocyte 3T3‐L1 cells, and that chronic tissue inflammation is an important cause of IR induced by obesity [39]. Therefore, we investigated the effects of GPR50 on insulin signaling pathways. Insulin receptor activation contributes to IRS1 phosphorylation, which initiates downstream signaling. When IRS1 is phosphorylated, its downstream signaling capacity is decreased [9, 11]. According to our experimental results, a GPR50 deficiency may significantly activate the insulin signaling pathway in which IRS1 is involved. As shown in Fig. 5, high glucose and PA treatment reduced p‐IRS1 expression by nearly 30% in normal 3T3‐L1 cells; however, high glucose and PA treatment did not downregulate p‐IRS1 expression in GPR50 knockout cells. These results indicate that GPR50 can effectively regulate IRS1 phosphorylation. We also found that a GPR50 deficiency activated p‐AKT, and although stimulation with high glucose and PA strongly activated p‐AKT, in GPR50 knockout 3T3‐L1 cells, p‐AKT was further activated. These results indicate that GPR50 might mediate the p‐IRS1/AKT pathway.

As shown in Figs 5 and 7, stimulation by insulin increased phosphorylation levels at AKT and insulin receptor substrate (IRS)1, but p‐IRS‐1 and p‐AKT were significantly decreased in the 3T3‐L1 cell line with GPR50 deficiency. However, with stimulation by PA or high glucose, p‐IRS‐1/IRS‐1 and p‐AKT/AKT levels increased significantly in GPR50^−/−^‐3T3‐L1 cells. We found that the IRS1/AKT pathway showed opposite results under different stimulations (insulin and PA or glucose).

Regarding this problem, we believe that when high concentrations of PA or glucose are stimulated, the main purpose is to activate metabolic pathways related to glucose metabolism and fatty acid synthesis, which mainly contributes to the synthesis of triglycerides, while the main function of insulin is to maintain the balance of blood glucose. They play different roles. As a receptor of unknown function, GPR50 regulates insulin signaling in response to various stimuli (Insulin and PA or Glucose) by regulating the IRS1/AKT pathway, indicating that it has a regulatory function for insulin signaling.

PPAR‐γ regulates the transcription of multiple genes involved in adipose precursor cell differentiation, regulates glucose uptake mediated by insulin, and increases insulin sensitivity. PPAR‐γ is closely related to inflammation and insulin resistance [40]. PPAR‐γ is important in regulating inflammatory cytokines. Downregulation of PPAR‐γ could strongly contribute to the effects of inflammatory cytokines on adipocytes. Additionally, by modulating IRS1 expression, PPAR‐γ plays an important role in the insulin signaling pathway [13]. The PPAR‐γ agonists fenofibrate and saroglitazar can improve IR [41, 42]. Induction of PPAR‐γ expression in adipocytes decreased IR in obese mice in a previous study [43].

In this study, we found that knockout of GPR50 significantly inhibited PPAR‐γ expression, suggesting that GPR50 is conducive to the activation of PPAR‐γ with increased plasma levels of free fatty acids (FFAs). PPAR‐γ activation in adipose tissue promotes lipid uptake and storage through the induction of target genes, including aP2, LPL, and CD36. PPAR‐γ also influences the effects of inflammatory cytokines and adipokines, including adiponectin, MCP‐1, and resistin. In addition to modulating the expression of adipokines, these changes can have beneficial effects on systemic glucose metabolism, including suppressing hepatic glucose uptake and stimulating skeletal muscle glucose uptake. Considering the roles that inflammation, the insulin signaling pathway, and PPAR‐γ expression play in the pathogenesis of insulin resistance, we tentatively suggest that GPR50 is involved in regulating inflammation and insulin signaling; therefore, GPR50 may contribute positively to insulin resistance.

## Conclusions

**Fig. 8:**
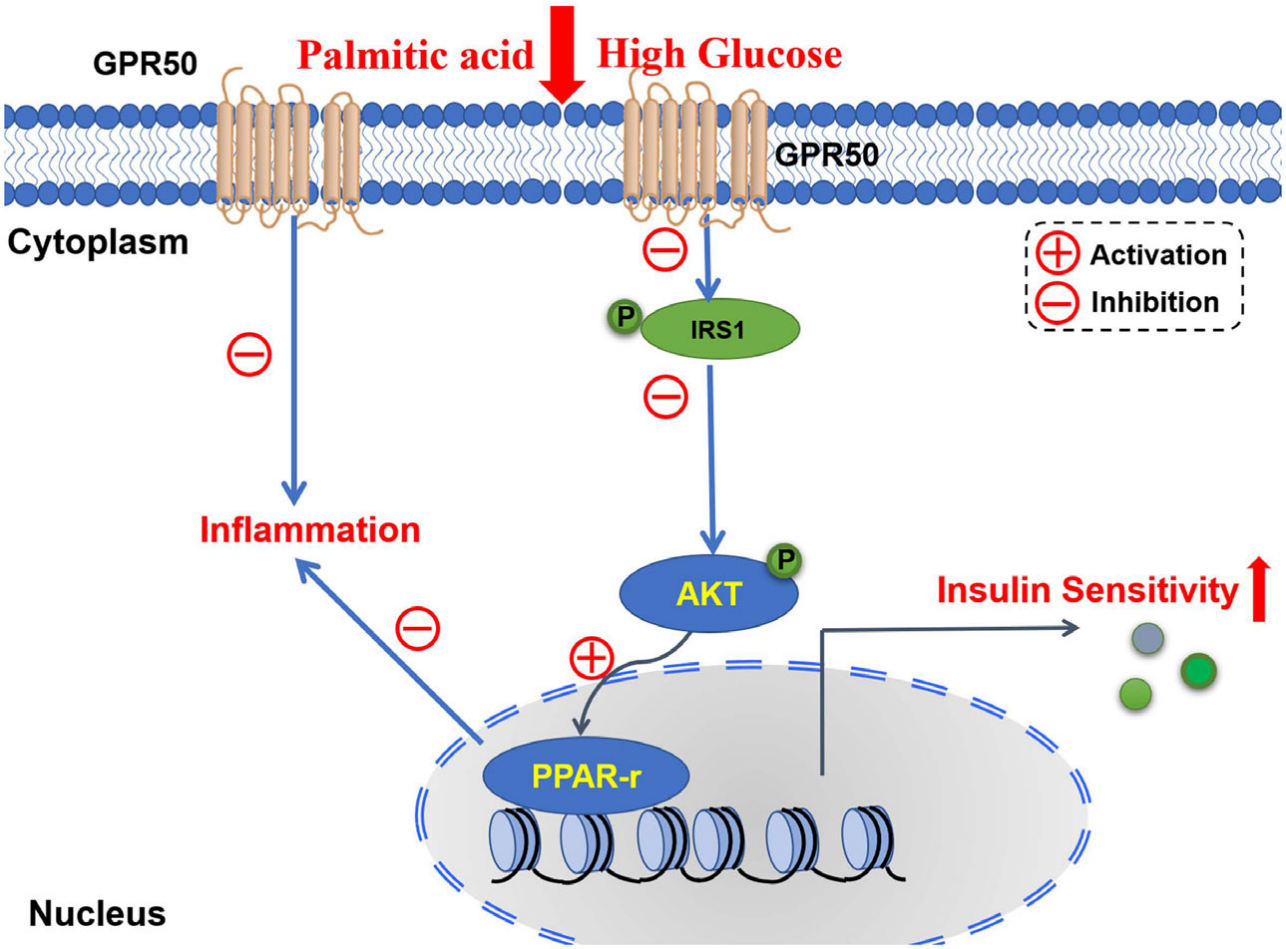
Hypothesized working model for GPR50‐mediated inflammation and insulin signaling regulation in the 3T3‐L1 cell line. GPR50 inhibits inflammation in adipose tissue and improves insulin signaling induced by high glucose or palmitic acid by inhibiting the phosphorylation of IRS1 and AKT. As a result, GPR50 might improve inflammation and insulin signaling and promote PPAR‐γ expression.

We identified GPR50 as a novel candidate for IR targeting in adipocytes. We demonstrate that knockout of GPR50 in 3T3‐L1 cells increases inflammation levels and insulin signaling pathway. Insulin resistance is mediated by the IRS1/AKT signaling pathway and PPAR‐γ expression. In conclusion, the above results suggest that GPR50 can improve inflammation and regulate insulin resistance through the regulation of the insulin signaling pathway, which may be accomplished by targeting PPAR‐γ transcription. (Fig. 8).

## Conflict of interest

The authors declare no conflict of interest.

## Author contributions

ZY was involved in data curation; ZY, JM, JL, LL, WQ, and CL were involved in formal analysis; JVZ was involved in funding acquisition; ZY, JM, JL, and CL were involved in investigation; LL and WQ were involved in investigation; ZY and P‐GR were involved in writing—original draft; P‐GR was involved in writing—review & editing.

## Supporting information


**Fig. S1.** Pathological changes in chow and HFD mice about obesity and T2DM. (A) Body weight. (B) Representative images of the H&E‐stained adipose tissue of the mice. Scale bars 50 μm. (C) GTT. (D) Fast glucose level of HFD and CHOW groups mice. Data were analyzed using *T* test. The results are representative of three independent experiments. The values in bar graphs are means ± SD. *P < 0.05; **P < 0.01.
**Fig. S2.** GPR50 inhibits inflammation in the 3T3‐L1 cell line. The GPR50 overexpression 3T3‐L1 cells were stimulated with high glucose (16.7 mm) and PA (0.25 mm) for 48 h. The relative mRNA expression levels of MCP‐1 were analyzed by Q‐PCR; Data were analyzed using a two‐way ANOVA followed by Dunnett's multiple comparisons test. The results are representative of three independent experiments. Values in bar graphs are the mean ± SEM. ****P < 0.0001 compared with Control, ####*P* < 0.0001 compared with Control + Glucose, $$$$*P* < 0.0001 compared with Control + PA.

## Data Availability

Microarray and sample annotation data were deposited in the Gene Expression Omnibus under accession number GSE100028. The direct link to the deposited data is available at https://www.ncbi.nlm.nih.gov/geo/query/acc.cgi?acc=GSE100028. This is from our earlier study: DOI: 10.1002/oby. And the data openly available in a public repository.
