# Supplementary material for: Orphan receptor GPR50 attenuates inflammation and insulin signaling in 3T3‐L1 preadipocytes
Source: FEBS Open Bio. 2022 Dec 13;13(1):89–101. doi: 10.1002/2211-5463.13516 (PMC9811602; doi:10.1002/2211-5463.13516)
Supplement: Supplementary file 1 — Fig. S1. Pathological changes in chow and HFD mice about obesity and T2DM. (A) Body weight. (B) Representative images of the H&E‐stained adipose tissue of the mice. Scale bars 50 μm. (C) GTT. (D) Fast glucose level of HFD and CHOW groups mice. Data were analyzed using T test. The results are representative of three independent experiments. The values in bar graphs are means ± SD. *P < 0.05; **P < 0.01. Fig. S2. GPR50 inhibits inflammation in the 3T3‐L1 cell line. The GPR50 overexpression 3T3‐L1 cells were stimulated with high glucose (16.7 mm) and PA (0.25 mm) for 48 h. The relative mRNA expression levels of MCP‐1 were analyzed by Q‐PCR; Data were analyzed using a two‐way ANOVA followed by Dunnett's multiple comparisons test. The results are representative of three independent experiments. Values in bar graphs are the mean ± SEM. ****P < 0.0001 compared with Control, ####P < 0.0001 compared with Control + Glucose, $$$$P < 0.0001 compared with Control + PA. [file FEB4-13-89-s001.docx]

**Supporting Material**

The experiments related to HFD mice have been analyzed in detail by our group in published articles（[DOI: 10.1002/oby.22590](file:///C:\Users\Administrator\Desktop\ShareX.lnk)）, so these studies have not been put back in this article, and the results and methods are as follows.


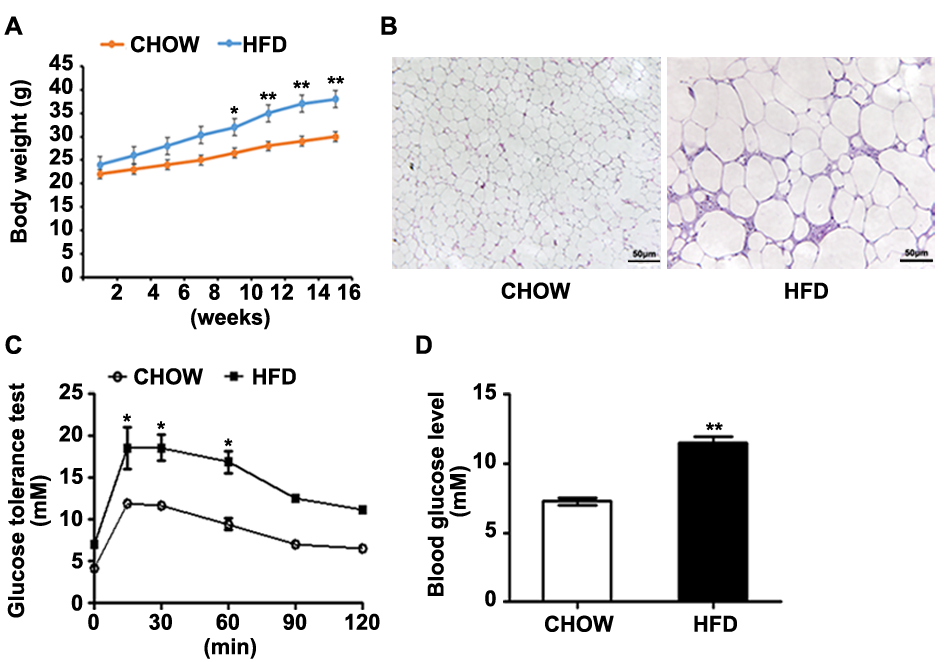


**Figure S1**: Pathological changes in chow and HFD mice about obesity and T2DM. (A) Body weight. (B) Representative images of the H&E-stained adipose tissue of the mice. Scale bars 50μm. (C) GTT. (D) Fast glucose level of HFD and CHOW groups mice. Data were analyzed using T test. The results are representative of three independent experiments. The values in bar graphs are means ± SD. ∗p < 0.05; **P < 0.01.

**
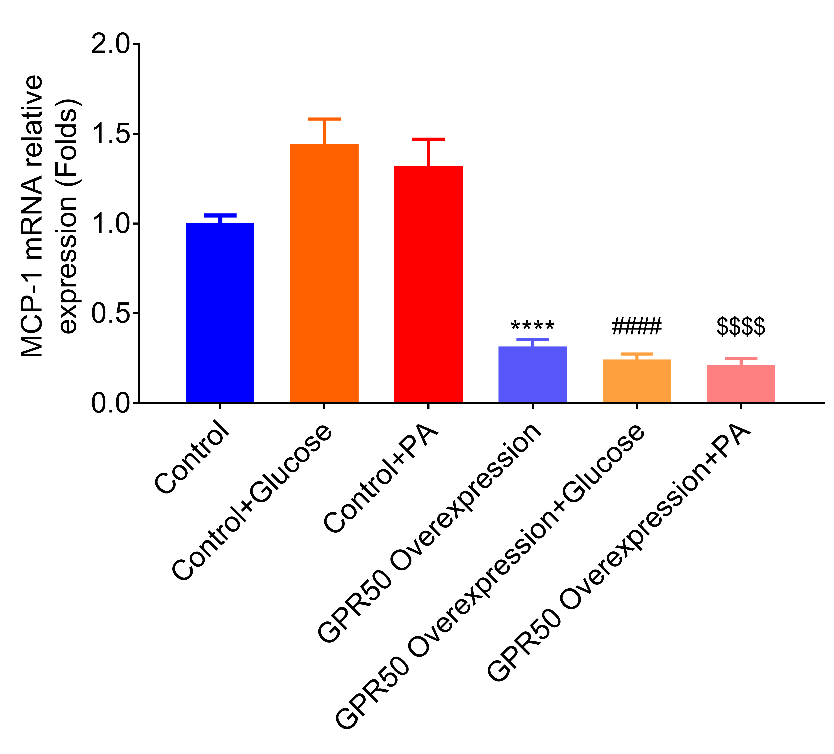
**

**Figure S2.** GPR50 inhibits inflammation in the 3T3L1 cell line. The GPR50 overexpression 3T3-L1 cells were stimulated with high glucose (16.7 mM) and PA (0.25 mM) for 48 h. The relative mRNA expression levels of MCP-1 were analyzed by Q-PCR; Data were analyzed using a two-way ANOVA followed by Dunnett's multiple comparisons test. The results are representative of three independent experiments. Values in bar graphs are the mean ± SEM. ****p <0.0001 compared with Control, ####p <0.0001 compared with Control + Glucose, $$$$p <0.0001 compared with Control + PA.
